# Interplay of population mobility, healthcare resources, and spatiotemporal clustering: epidemiology and prevention strategies for HIV among blood donors in Zhejiang, China

**DOI:** 10.3389/fpubh.2025.1666694

**Published:** 2025-10-02

**Authors:** Danxiao Wu, Jie Dong, Yaling Wu, Xiaotao Li, Guangshu Yu, Wenhong Wang, Jinhui Liu

**Affiliations:** Department of Laboratory, Blood Center of Zhejiang Province, Hangzhou, China

**Keywords:** blood safety, HIV epidemiology, spatiotemporal clustering, population mobility, healthcare resources

## Abstract

**Background:**

This study leverages Zhejiang Province’s HIV-confirmed positive blood donor database (2017–2024), integrating multidimensional data including demographic, serological, geospatial, and policy indicators to systematically analyze infection risk factors, screening marker characteristics, spatiotemporal distribution patterns, migration impacts, and the regulatory effects of healthcare resource allocation and government investment on donor HIV positivity.

**Methods:**

5,204,965 voluntary donors underwent nucleic acid/serological testing. Multivariate logistic regression, spatiotemporal scan statistics (SaTScan), estimated annual percentage change (EAPC) modeling, and correlation analyses were applied. Healthcare capacity was evaluated via principal component analysis (PCA) index; future trends projected using autoregressive integrated moving average (ARIMA).

**Results:**

During April 2017 to December 2024, 449 HIV-positive donors were confirmed (8.63 per 100,000 donors), with significant risk factors including people moving in from high-prevalence areas (OR 1.56), male gender (OR 7.32), self-employed (OR 1.46), and non-regular donation status (OR 1.86), while older age (OR 0.97) and government employment (OR 0.49) served as protective factors. Among confirmed positives, 98.44% exhibited HIV Ag⁺Ab⁺NAT⁺ reactivity. There was significant provincial decline in positivity (EAPC = −12.41, *p* < 0.001) with March–July seasonal peak (*p* = 0.017) and spatial cluster in northeastern Zhejiang during March 2018 (p < 0.001). The monthly HIV-positive rate among blood donors was significantly correlated with general population AIDS incidence (r = 0.445, *p* < 0.001). Age-gender disparities profiling revealed peak male positivity among 21-25-year-olds concentrated in northern Zhejiang, while females aged 46–50 showed the highest burden in eastern Zhejiang. Migration analysis indicated 31.02% (125/403) of HIV-positive donors originated from 10 high-incidence provinces from 2018 to 2024, and influx correlated with birthplace-specific positivity (*p* < 0.001). Healthcare capacity (*p* = 0.014) and government health expenditure (*p* = 0.034) were both inversely correlated with donor positivity. ARIMA projections for 2025–2030 indicate oscillating declines in overall and male donors, while female rates stabilize.

**Conclusion:**

Centralized testing and cross-regional deferral strategies have significantly reduced HIV positivity among Zhejiang’s donors. Persistent challenges include window-period transmission, low-viremia infections under antiretroviral therapy. Further reduction of residual transfusion risks requires integrated epidemiological surveillance, high-risk population interventions, and optimized healthcare resource allocation.

## Introduction

Blood transfusion therapy serves as a critical supportive measure in surgical procedures, hematological diseases, and obstetric emergencies, playing an irreplaceable role in clinical medicine (1–3). However, the risk of transfusion-transmitted infections (TTIs) remains a core challenge in blood safety, with human immunodeficiency virus (HIV) being a global public health priority due to its prolonged latency period, diagnostic window limitations, and incurability (4, 5). According to UNAIDS, approximately 39.9 million people globally were living with HIV in 2023, with 1.3 million new infections reported (6). While China has significantly reduced the residual risk of HIV transmission through blood transfusions since implementing nationwide nucleic acid testing (NAT) in 2016, emerging challenges stemming from regional epidemiological disparities and population mobility require further investigation (7–9).

Zhejiang Province, as an economically developed region in eastern China, maintains a relatively low overall HIV prevalence. However, infection dynamics among blood donors exhibit complex characteristics. Among the HIV-infected population in Zhejiang Province, the majority of cases are associated with men who have sex with men (MSM) (10), or having multiple sexual partners (11, 12), highlighting evolving transmission patterns in high-risk populations. Although China’s mandatory donor screening strategies which combined antibody testing and NAT have substantially mitigated transfusion risks (13), potential threats persist due to window-period infections, biomarker diversity, and population migration (14, 15).

Current research predominantly focuses on HIV trends, lacking in-depth regional analyses of blood donor population dynamics, particularly comprehensive assessments integrating demographic structures, diagnostic biomarker characteristics, and spatiotemporal cluster risks. This study leverages Zhejiang Province’s 2017–2024 HIV-confirmed blood donor database to systematically analyze infection risk factors, screening biomarker profiles, spatiotemporal distribution patterns, migration impacts, and the regulatory effects of healthcare resource allocation and government investment on HIV positivity rates. By employing decomposition models and machine learning to predict trends in donor HIV positivity, we aim to provide empirical evidence for optimizing regional blood screening protocols and reducing residual transfusion transmission risks.

## Materials and methods

### Blood specimens and donor information

Voluntary, non-remunerated blood donor specimens were collected from 12 stations across 11 Zhejiang Province regions between April 2017 and December 2024. Since both the Jinhua and Yiwu blood stations are located within Jinhua City, their data were merged. Additionally, the regions of Zhejiang were divided into five areas: Northern Zhejiang, Eastern Zhejiang, Central Zhejiang, Southern Zhejiang, and Northwestern Zhejiang (Supplementary Table 1). Donors underwent pre-donation and post-donation screening according to regulations established by the Chinese government (13, 16). All specimens were collected, stored, and processed following the manufacturer’s instructions.

All 12 blood service institutions in Zhejiang Province utilize the same blood information management system approved by the Zhejiang Provincial Health Administration, uniquely identifying donors via national ID numbers. In this system demographic data were recorded. If a donor donated blood twice or more within a year, they were counted as one donor for analysis purposes. Information on HIV-positive donors was obtained from the Zhejiang Province HIV Confirmed Positive Donor Deferral Registry Database. Furthermore, a cross-regional donor deferral system was in effect, built upon this registry. A province-wide database of permanently deferred donors (including all HIV-confirmed positives) was maintained, and all blood stations were required to check prospective donors against this database prior to donation, effectively preventing any previously identified positive donor from donating again anywhere in the province. Informed consent was obtained from all donors.

### HIV serological, nucleic acid screening, and confirmation testing

Blood stations used enzyme-linked immunosorbent assay (ELISA) kits from two different manufacturers for HIV antibody or antigen screening tests. These included Beijing Wantai (Beijing, China), Zhuhai Livzon (Zhuhai, China), InTec Products (Xiamen, China), and BIO-RAD Laboratories (Hercules, CA, USA). HIV nucleic acid testing (NAT) was performed using 6-pooled (Roche Molecular Systems, Inc., Branchburg, NJ, USA), 8-pooled (Haoyuan Biotech Co., Ltd., Shanghai, China), and individual donation (ID) testing (Grifols Diagnostic Solutions Inc., Emeryville, CA, USA). The specific screening methods and reagents used depended on the choices of each individual blood station. Although pooling strategies varied, all NAT testing complied with national sensitivity standards. All testing procedures strictly followed the manufacturers’ instructions. A specimen was considered screening test reactive if any reagent test result was reactive (13).

Specimens reactive in HIV screening tests at the blood stations were sent to the local Center for Disease Control and Prevention (CDC) in China for confirmation (17). Donors were classified as HIV confirmed positive if the CDC confirmed positivity via Western blot assay and/or a positive viral load test (HIV RNA detection). Otherwise, donors were considered HIV confirmed negative. Therefore, potential variations in sensitivity among different screening kits could influence the initial reactivity rate but would not bias the confirmed positivity rate that serves as the basis for all our subsequent analyses.

### HIV positivity rate and age-standardized positivity rate

The HIV Positivity Rate was calculated by dividing the number of confirmed HIV-positive donors within a given time period (e.g., year or month) by the total number of donors in that period, expressed per 100,000 donors. The age-standardized positivity rate (ASPR) was calculated using direct standardization, adjusted to the provincial donor population demographics from 2017 to 2024 to correct for demographic differences (Supplementary Table 2).

### Estimated annual percentage change (EAPC) calculation

To analyze trends in the age-standardized rate (ASR) of HIV positivity, we employed the EAPC method. This method involves a regression model expressed as (18):


ln(ASR)=α+βX+ε

In this equation, the natural logarithm of the age-standardized rate is represented as ln(ASR). X represents the calendar year under consideration. The intercept is denoted by α, and β represents the slope, indicating the trend over time. Any error in the model is represented by ε. The EAPC is expressed as 100 × [exp(β) − 1], representing the annual percentage change. We used the EAPC and its 95% confidence interval (CI) to analyze the trend.

### Decomposition analysis

A decomposition analysis based on the method developed by Das Gupta (19) was performed to quantify the driving factors behind changes in the HIV positivity rate. Specifically, we decomposed the change in HIV Positivity Rate from 2018 to 2024 into three explanatory components: changes due to population growth, changes in population structure by sex, and epidemiological changes.

### Autoregressive integrated moving average (ARIMA) time series forecasting model

ARIMA consists of three main components: autoregressive (AR), integrated (I), and moving average (MA). ARIMA models are typically denoted as ARIMA (p, d, q), where *p* is the order of the autoregressive component, d is the degree of differencing (integration), and q is the order of the moving average component (20). Based on the HIV positivity rate time series data from 2018 to 2024, the ARIMA model was used to forecast future trends in the HIV positivity rate for 2025–2030.

### Spatiotemporal epidemiological analysis and spatiotemporal scan cluster analysis

Vector maps of Zhejiang Province at the prefectural city and county (district) levels were downloaded from the National Geographic Information Public Service Platform as the base map. This map carries the official review certification from the Ministry of Natural Resources of the People’s Republic of China (MNR) under approval number GS(2024)0650. ArcGIS 10.2 software was applied for spatial autocorrelation analysis, calculating the global Moran’s I index and local Moran’s I index. SaTScan version 9.7 software was used for spatiotemporal scan analysis. The discrete Poisson model was used. The scanning time range was from January 1, 2018, to December 31, 2024, with a time interval of “month.” The maximum spatial cluster size was set to include up to 50% of the population at risk, with the “no geographical overlap” constraint to identify distinct, high-priority clusters for intervention. To assess the robustness of our cluster detection to parameter selection, we conducted sensitivity analyses by varying the maximum spatial cluster size (30 and 40%) and allowing geographical overlap (50% threshold). A cluster was considered statistically significant if the log-likelihood ratio (LLR) test yielded *p* < 0.05.

### Calculation of medical resource index in Zhejiang Province

A composite Medical Resource Index was constructed for each region using Principal Component Analysis (PCA) to reduce the dimensionality of four healthcare resource variables (all measured per 1,000 population), including the number of hospital beds, physicians, nurses, and healthcare technicians. Prior to analysis, all variables were standardized to ensure equal weighting. PC1 and PC2 together explained 98.5% of the total variance in the original variables (Supplementary Table 7a), providing a highly efficient summary of regional healthcare infrastructure. Thus, the Medical Resource Index was calculated as a weighted sum of PC1 and PC2 scores. The loadings of the original variables on these components, which indicate the correlation and contribution of each variable to the PC, are provided in Supplementary Table 7b.

### Statistical analysis

Data were processed using R 4.4.1 and STATA 17.0. Continuous variables used Mann–Whitney U test, and categorical variables used the Chi-square or Fisher’s exact test. All statistical tests were two-sided. Data are presented numerically with their 95% confidence intervals (CIs) or 95% uncertainty intervals (UIs). Pearson correlation analysis was selected to assess linear correlations between continuous variables. The *p*-value < 0.05 was considered statistically significant.

## Results

### HIV screening markers and Western blot banding patterns in confirmed positive donors

The samples were categorized into four groups based on the NAT and serological screening results from blood stations (Table 1). Among 449 confirmed positive blood donors, 98.44% (442/449) tested HIV Ag^+^Ab^+^NAT^+^ during blood station screening, 0.22% (1/449) were HIV Ag^+^Ab^−^NAT^+^, 0.45% (3/449) were HIV Ag^−^Ab^−^NAT^+^, and 0.45% (3/449) were HIV Ag^+^Ab^+^NAT^−^. Notably, 62.58% (281/449) lacked Western blot (WB) banding information. Among these 281 donors, 3 were NAT-positive but lacked WB results, and the remainder were HIV-1 infections.

**Table 1:** HIV screening markers and western blot banding patterns in confirmed positive donors.

Screening test result^@^	Ag^+^Ab^+^NAT^+^	Ag^+^Ab^+^NAT^−^	Ag^+^Ab^−^NAT^+^	Ag^−^Ab^−^NAT^+^
Detected band (*n* = 168)	**163**	**2**	**0**	**3**
gp160 + gp120 + gp41 + p24 + p17 + p55 + p66 + p51 + p31	34	2	0	0
gp160 + gp120 + gp41 + p24 + p17 + p66 + p51 + p31	51	0	0	0
gp160 + gp120 + gp41 + p24 + p55 + p66 + p51 + p31	3	0	0	0
gp160 + gp120 + gp41 + p24 + p17 + p55 + p66 + p51	2	0	0	0
gp160 + gp120 + gp41 + p24 + p66 + p51 + p31	14	0	0	0
gp160 + gp120 + gp41 + p24 + p17 + p66 + p51	2	0	0	0
gp160 + gp120 + gp41 + p24 + p17 + p66 + p31	2	0	0	0
gp160 + gp120 + gp41 + p24 + p66 + p55 + p31	1	0	0	0
gp160 + gp120 + gp41 + p24 + p17 + p31	17	0	0	0
gp160 + gp120 + gp41 + p24 + p66 + p51	5	0	0	0
gp160 + gp120 + gp41 + p24 + p17 + p66	3	0	0	1
gp160 + gp120 + gp41 + p24 + p31 + p66	1	0	0	0
gp160 + gp120 + gp41 + p24 + p31	18	0	0	1
gp160 + gp120 + gp41 + p24 + p66	2	0	0	0
gp160 + gp120 + gp41 + p24 + p17	1	0	0	0
gp160 + gp120 + gp41 + p31	1	0	0	0
gp160 + gp120 + p24 + p31	1	0	0	0
gp160 + gp41 + p24	4	0	0	1
gp160 + p24	1	0	0	0
Band report not provided (*n* = 281)	**279**	**1**	**1**	**0**
HIV 1 type	276	1	1	0
NAT positive	3	0	0	0
Total Number (*n* = 449)	**442**	**3**	**1**	**3**

^@^Samples were classified based on the results of the Ag/Ab ELISA testing and nucleic acid screening at the blood center. Bold values indicate a statistically significant difference.

Of the 37.42% (168/449) with documented WB banding patterns, 21.43% (36/168) exhibited full banding patterns including gp160, gp120, gp41, p24, p17, p55, p66, p51, and p31. Notably, two HIV Ag^+^Ab^+^NAT^−^ samples also demonstrated full banding patterns (Table 1). The three HIV Ag^−^Ab^−^NAT^+^ donors required extended follow-up periods (57, 57, and 124 days, respectively) for confirmation. Their WB patterns emerged as follows: gp160 + gp120 + gp41 + p24 + p17 + p66, gp160 + gp120 + gp41 + p24 + p31, and gp160 + gp41 + p24 (Table 1; Supplementary Figure 1). Across all WB patterns, the absence of p55, p51, and p17 bands was most frequently observed (Table 1; Supplementary Figure 1).

### Analysis of baseline characteristics and risk factors among HIV-confirmed positive blood donors

Since the implementation of the HIV confirmed positive donor deferral database in Zhejiang Province in April 2017, a total of 449 confirmed HIV-positive donors were registered through December 2024, yielding an infection rate of 8.63 per 100,000 blood donors (449/5,204,965). The recorded demographic variables included age, body mass index (BMI), native place, gender, ABO and RhD blood types, nationality, marital status, education level, occupation, types of donors, blood donation type, interval time of donation (days), and times of donation. A donor’s native place was classified as a “high-prevalence province” if it was among the top 10 provinces with the highest average annual reported AIDS incidence rates in China from 2018 to 2023, according to the China Health Statistics Yearbooks (Table 2; Supplementary Table S6).

**Table 2:** Univariate and multivariate analyses of basic clinical characteristics related to blood donors to determine the risk factors for HIV infection.

Basic clinical characteristics^@^	Total (*n* = 5,204,965)	HIV Non-infection (*n* = 5,204,516)	HIV infection (*n* = 449)	*p*-value^#^	Univariable Analysis		**Mutivariable Analysis** ^**&**^	
					OR (95% CI)	*p*-value	OR (95% CI)	*p*-value
Age, M (Q1, Q3)	34 (25, 43)	34 (25, 43)	30 (24, 40)	**<0.001**	0.979287(0.970548–0.987993)	**<0.001**	0.970165(0.957802–0.982697)	**<0.001**
BMI, x̄ ± s	24.63 ± 3.46	24.63 ± 3.46	24.35 ± 3.37	0.088	0.976366(0.949628–1.003263)	0.088		
Native place, *n* (%)*				**<0.001**				
Lower prevalence areas (Ref)	4,269,186(82.02)	4,268,877(82.02)	309(68.82)					
Higher prevalence areas	935,779(17.98)	935,639(17.98)	140(31.18)		2.067206(1.688471–2.518234)	**<0.001**	1.563039(1.270439–1.913877)	**<0.001**
Gender, *n* (%)				**<0.001**				
Female (Ref)	1,994,459(38.32)	1,994,420(38.32)	39(8.69)					
Male	3,210,506(61.68)	3,210,096(61.68)	410(91.31)		6.531766(4.769429–9.215762)	**<0.001**	7.324400(5.342230–10.343750)	**<0.001**
ABO blood type, *n* (%)				0.792				
A (Ref)	1,581,041(30.38)	1,580,908(30.38)	133(29.62)					
B	1,348,044(25.9)	1,347,919(25.9)	125(27.84)		1.103841(0.894998–1.352856)	0.348		
O	1,837,106(35.3)	1,836,954(35.3)	152(33.85)		0.938218(0.769917–1.138605)	0.523		
AB	438,774(8.43)	438,735(8.43)	39(8.69)		1.033268(0.732331–1.415086)	0.845		
RhD blood type, *n* (%)				0.741				
RhD^−^ (Ref)	28,640(0.55)	28,637(0.55)	3(0.67)					
RhD^+^	5,176,325(99.45)	5,175,879(99.45)	446(99.33)		0.822547(0.315589–3.316341)	0.736		
Nationality, *n* (%)				0.615				
Chinese Nationality (Ref)	5,193,916(99.79)	5,193,468(99.79)	448(99.78)					
Foreign Nationality	11,049(0.21)	11,048(0.21)	1(0.22)		1.049268(0.059774–4.634138)	0.962		
Marital Status, *n* (%)				**<0.001**				
Married (Ref)	1,393,064(26.76)	1,392,957(26.76)	107(23.83)					
Unmarried	2,168,174(41.66)	2,167,941(41.65)	233(51.89)		1.510926(1.255672–1.818857)	**<0.001**	0.979607(0.729471–1.320665)	0.892
Other	1,643,727(31.58)	1,643,618(31.58)	109(24.28)		0.694549(0.557404–0.858541)	**0.001**	1.232782(0.918279–1.653454)	0.163
Education level, *n* (%)				**<0.001**				
Primary school (Ref)	101,027(1.94)	101,014(1.94)	13(2.9)					
Junior high school	949,379(18.24)	949,276(18.24)	103(22.94)		1.334430(1.065958–1.655962)	**0.010**	1.370118(1.057730–1.767388)	**0.016**
Middle school	972,393(18.68)	972,321(18.68)	72(16.04)		0.831277(0.641157–1.062336)	0.151		
College	941,669(18.09)	941,588(18.09)	81(18.04)		0.996515(0.778362–1.260139)	0.977		
Undergraduate	1,096,104(21.06)	1,096,043(21.06)	61(13.59)		0.589313(0.445698–0.765505)	**<0.001**	0.823879(0.605589–1.106379)	0.207
Graduate and above	89,866(1.73)	89,862(1.73)	4(0.89)		0.511608(0.158305–1.195487)	0.182		
Other	1,054,527(20.26)	1,054,412(20.26)	115(25.61)		1.355200(1.092155–1.669338)	0.005	1.196632(0.935360–1.526020)	0.150
Occupation, *n* (%)				**0.001**				
Military (Ref)	77,323(1.49)	77,321(1.49)	2(0.45)					
Government employee	241,734(4.64)	241,725(4.64)	9(2.00)		0.419941(0.200713–0.763173)	**0.010**	0.494383(0.233473–0.914781)	**0.041**
Medical staff	334,301(6.42)	334,288(6.42)	13(2.90)		0.434389(0.237358–0.721951)	**0.003**	0.751087(0.405903–1.267777)	0.321
Student	511,914(9.84)	511,873(9.84)	41(9.13)		0.921252(0.658316–1.253402)	0.617		
Clerk	896,472(17.22)	896,380(17.22)	92(20.49)		1.238566(0.979531–1.549965)	0.067		
Farmer	254,248(4.88)	254,227(4.88)	21(4.68)		0.955396(0.597438–1.441306)	0.838		
Worker	389,323(7.48)	389,280(7.48)	43(9.58)		1.310091(0.943292–1.771597)	0.092		
Sole Trader	48,648(0.93)	48,642(0.93)	6(1.34)		1.435617(0.568194–2.929302)	0.379		
Self-employed	433,786(8.33)	433,736(8.33)	50(11.14)		1.378365(1.015090–1.830085)	**0.032**	1.457541(1.063520–1.956541)	**0.015**
Other	2,017,216(38.76)	2,017,044(38.76)	172(38.31)		0.981251(0.809962–1.185405)	0.845		
Types of donors, *n* (%)				**<0.001**				
Regular blood donors (Ref)	1,357,546(26.08)	1,357,511(26.08)	35(7.80)					
Non-regular blood donors	3,847,419(73.92)	3,847,005(73.92)	414(92.20)		4.174096(3.002990–6.000088)	**<0.001**	1.857436(1.252798–2.847356)	**0.003**
Donation type, *n* (%)				**<0.001**				
Platelet (Ref)	751,001(14.43)	750,987(14.43)	14(3.12)					
Whole blood	4,453,964(85.57)	4,453,529(85.57)	435(96.88)		5.239605(3.204622–9.366254)	**<0.001**	3.471322(1.909258–6.895030)	**<0.001**
Interval time of donation (days), M (Q1, Q3)	553 (254, 2,622)	552 (254, 2,621)	2,493(650, 5,606)	**<0.001**	1.000121(1.000100–1.000141)	**<0.001**	1.000122(1.000099–1.000142)	**<0.001**
Times of donation, M (Q1, Q3)	2 (1, 6)	2 (1, 6)	1 (1, 2)	**<0.001**	0.934710(0.915012–0.956307)	**<0.001**	1.000004(0.984565–1.010401)	1.000

^@^The normal distribution of numerical variables is represented by the mean ± standard deviation (x̄  ±  s), non-normal distributions are represented by the median [first quartile, third quartile; M (Q1, Q3)] and categorical variables are represented by frequency [percentage; *n* (%)]. ^*^The determination of high and low prevalence areas is based on China Health Statistics Yearbook (2018–2023). The high prevalence areas refer to the 10 provinces/municipalities with the highest AIDS incidence rates, including Sichuan, Guangxi, Chongqing, Guizhou, Yunnan, Xinjiang, Hunan, Guangdong, Jiangxi, and Henan. Refer to Supplementary Table S6 for details. ^#^Bivariate analyses were conducted using the chi-square test, the Fisher exact test or the Wilcoxon rank-sum test; the p-value was derived from bivariate association analyses between each study variable and the HIV infection state. (ref), Reference category for odds ratio (OR) calculation. ^&^Only variables that remained statistically significant (*p* < 0.05) in the multivariable model are presented with ORs and 95% CIs. Bold values indicate a statistically significant difference.

Comparative analysis revealed significant differences in demographic distributions between HIV-infected and non-infected donors regarding age, native place, gender, marital status, education level, occupation, types of donors, blood donation type, interval time of donation (days), and times of donation (Table 2). Univariate analysis identified statistically significant associations with the following variables: age, native place, gender, unmarried and other marital status, junior high school and undergraduate education level, occupation including government employee, medical staff and self-employed, types of donors, blood donation type, interval time of donation (days), and times of donation (Table 2; Supplementary Figure 2A).

Multivariate analysis demonstrated that independent risk factors for HIV infection included native place in high-prevalence regions (OR 1.563039, 95% CI 1.270439–1.913877, see Supplementary Table S6 for the list of provinces), male gender (OR 7.324400, 95% CI 5.342230–10.343750), junior high school education level (OR 1.370118, 95% CI 1.057730–1.767388), self-employment (OR 1.457541, 95% CI 1.063520–1.956541), non-regular blood donor (OR 1.857436, 95% CI 1.252798–2.847356), whole blood donation type (OR 3.471322, 95% CI 1.909258–6.895030), and extended donation intervals (OR 1.000122, 95% CI 1.000099–1.000142). Older age (OR 0.970165, 95% CI 0.957802–0.982697) and government employment (OR 0.494383, 95% CI 0.233473–0.914781) emerged as protective factors (Table 2; Supplementary Figure 2B).

### Geographic variations in annual HIV positivity rate across Zhejiang Province

**Figure 1:**
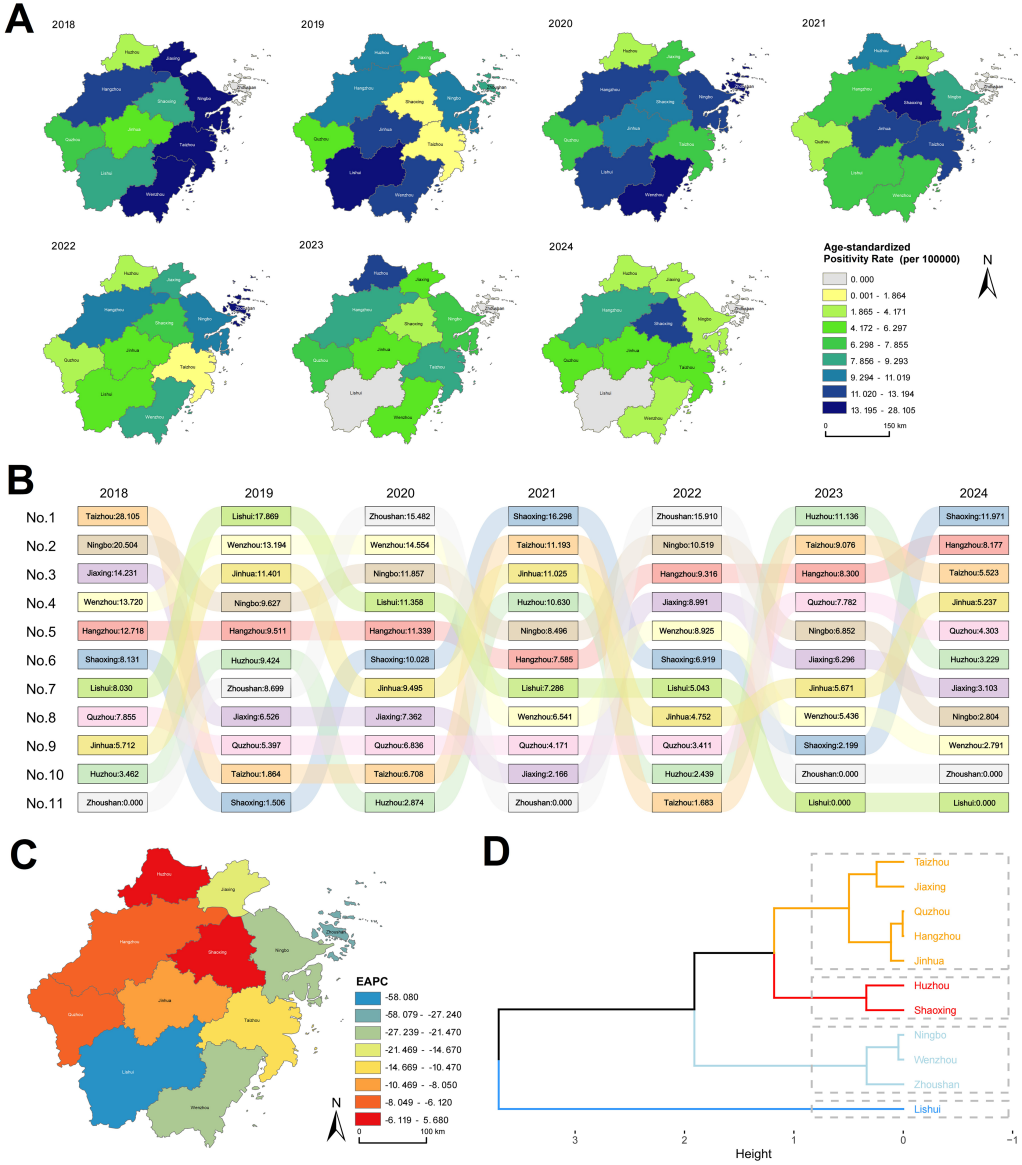
Annual changes in ASPR by region in Zhejiang Province, 2018–2024. **(A)** Distribution map of ASPR by region in Zhejiang Province. **(B)** Ranked bar chart of ASPR by region in Zhejiang Province. **(C)** Estimated Annual Percentage Change (EAPC) map of ASPR by region in Zhejiang Province. **(D)** Cluster analysis map of ASPR EAPC by region in Zhejiang Province.

Given incomplete annual data prior to April 2017 when the database was initiated, analyses were restricted to the period spanning 2018 to 2024. The overall HIV annual positivity rate changes in Zhejiang Province demonstrated a significant declining trend (EAPC = −12.41, *p* < 0.001; Supplementary Table 3; Figure 1A). However, substantial fluctuations were observed in regional rankings (Figure 1B). For instance, in 2018, the three regions with the highest rates were Taizhou (28.105 per 100,000), Ningbo (20.504 per 100,000), and Jiaxing (14.231 per 100,000). By 2024, the highest rates shifted to Shaoxing (11.971 per 100,000), Hangzhou (8.177 per 100,000), and Taizhou (5.523 per 100,000). Notably, Ningbo declined to 8th position (2.804 per 100,000) and Jiaxing to 7th (3.103 per 100,000) in 2024. Shaoxing rose from 6th (8.131 per 100,000) in 2018 to 1st in 2024 with an absolute rate increase. Hangzhou advanced from 5th (12.718 per 100,000) to 2nd position by 2024, though its rate decreased.

Analysis of annual positivity rate changes using the EAPC method identified statistically significant declines in Wenzhou (*p* = 0.0064), Ningbo (*p* = 0.0132), and Lishui (*p* = 0.0176), while other regions showed non-significant trends (Supplementary Table 3; Figure 1C). Cluster analysis of EAPC values revealed the steepest decline in Lishui, followed by Ningbo, Wenzhou, and Zhoushan. Huzhou exhibited the smallest reduction, whereas Shaoxing displayed an increasing trend (Supplementary Table 3; Figure 1D).

### Temporal and spatial analysis of HIV positivity rate among blood donors in Zhejiang Province

**Figure 2:**
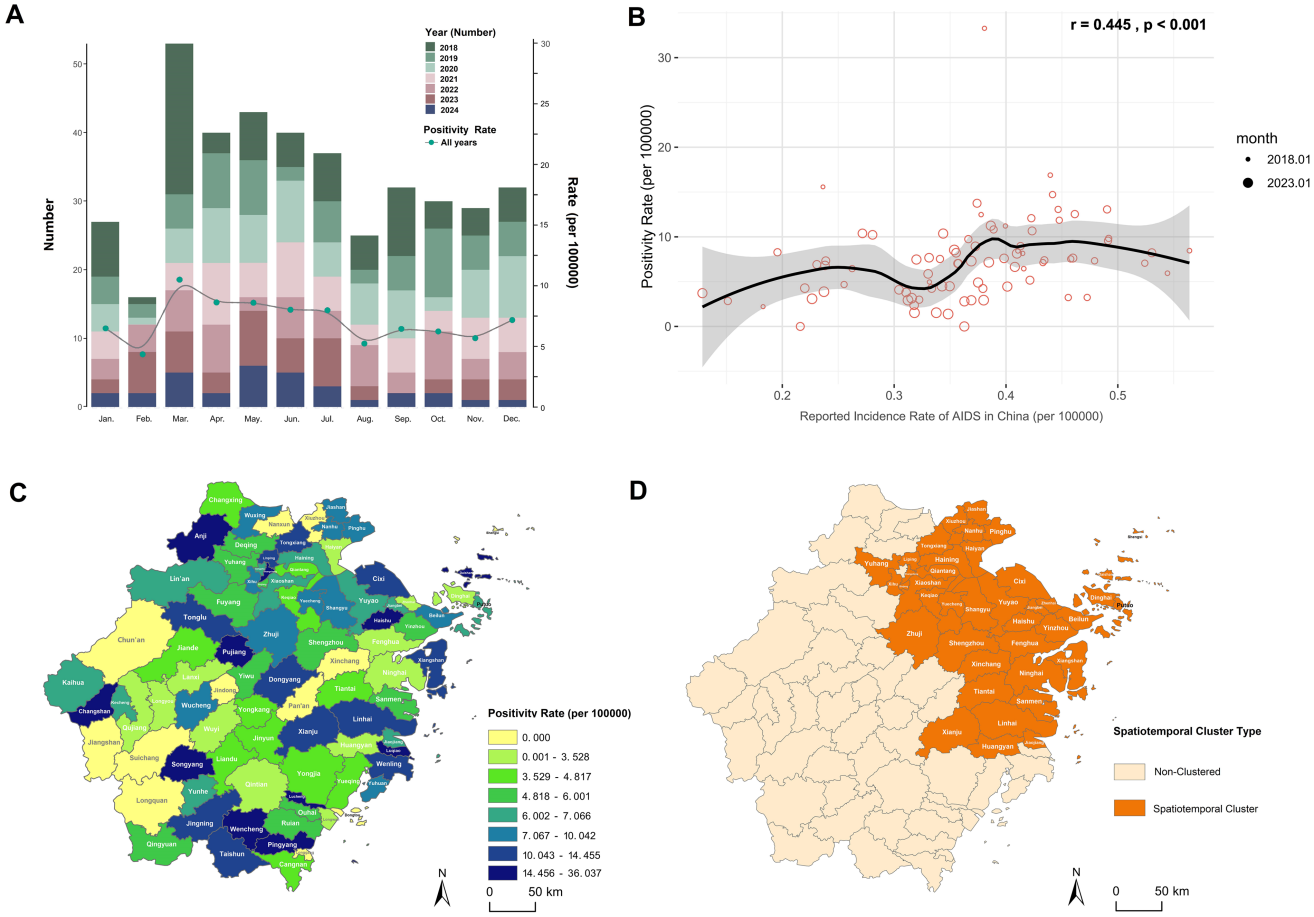
Spatiotemporal distribution characteristics of HIV positivity rate among blood donors in Zhejiang Province. **(A)** Monthly number of HIV-positive blood donors and HIV positivity rate in Zhejiang Province, 2018–2024. **(B)** Correlation analysis between the monthly HIV positivity rate among blood donors in Zhejiang Province and the monthly AIDS incidence rate reported by the China CDC, using Pearson correlation. The monthly incidence rate was calculated based on AIDS case numbers reported monthly by the China CDC (21): Monthly AIDS incidence rate (per 100,000 population) = (Number of reported AIDS cases in the month / Resident population of China at the end of the previous year, as published by the National Bureau of Statistics) × 100,000. **(C)** Distribution map of the total HIV positivity rate by county/district in Zhejiang Province, 2018–2024. **(D)** Spatiotemporal clustering areas for HIV positivity rate among blood donors in Zhejiang Province, March 2018.

Monthly analysis of HIV-positive cases and positivity rates among blood donors in Zhejiang Province from 2018 to 2024 revealed March as the peak month for positivity rates (Figure 2A). Seasonal scan analysis identified a statistically significant high-risk period from March to July (RR = 1.38, LLR = 5.748, *p* = 0.017). Correlation analysis between monthly HIV positivity rate in blood donors and monthly AIDS incidence rates reported by the Chinese Center for Disease Control and Prevention (21) demonstrated a significant positive association (r = 0.445, *p* < 0.001; Figure 2B). Although these two metrics differ in their calculation and population base, this correlation indicates that the temporal patterns captured in the donor screening data reflect broader epidemiological trends of HIV transmission in the general population.

Global spatial autocorrelation analysis of county-level positivity rates across the province yielded a Moran’s I index of 0.016 (Z-score = 0.413, *p* = 0.680), suggesting no statistically significant spatial clustering. This implies a random geographic distribution of HIV-positive donors without identifiable high- or low-incidence clusters (Figure 2C). Subsequent space–time scan analysis detected one statistically significant spatiotemporal cluster (Supplementary Table 4; Figure 2D), primarily concentrated in northeastern Zhejiang (such as Hangzhou, Ningbo, Taizhou, and Shaoxing) during March 2018. A sensitivity analysis demonstrated that this core finding was highly robust to changes in model parameters. The same spatiotemporal cluster (March 2018 in northeastern Zhejiang) remained the most statistically significant across all alternative parameter sets, including models with a more restrictive population threshold (30 and 40%) and a model allowing geographical overlap. While the precise spatial extent varied slightly with stricter thresholds, the identified high-risk area consistently centered on the cities of Hangzhou, Ningbo, Taizhou, and Shaoxing. The high relative risk estimates (RR range: 7.86–9.32) were also consistent across models (Supplementary Table 4a).

### Geographic, gender, and age-specific trends in HIV positivity rate among blood donors in Zhejiang Province

**Figure 3:**
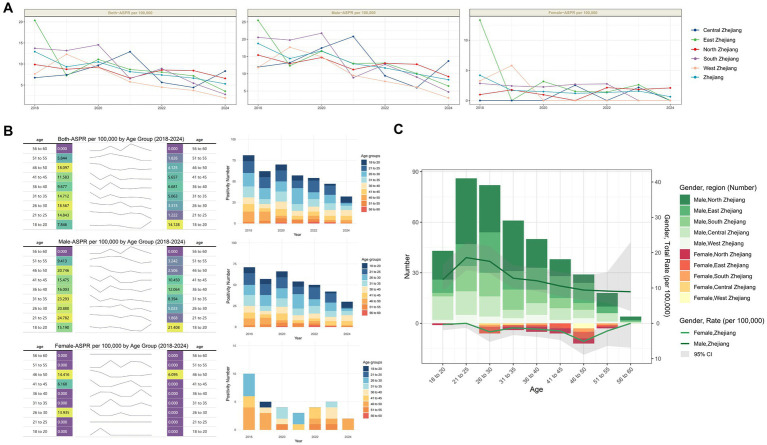
Age and Sex characteristics of HIV positivity rate among blood donors in Zhejiang Province. **(A)** Trends in positivity rate by sex across different regions of Zhejiang Province, 2018–2024. **(B)** Trends in HIV positivity rate and number by age group and sex in Zhejiang Province, 2018–2024. **(C)** Total number of positives and positivity rate by region, sex, and age group in Zhejiang Province (overall 2018–2024). The gray area reflects the 95% confidence interval of the positivity rate.

Initial analyses revealed elevated HIV positivity rates among younger male donors (Table 1; Supplementary Figure 2). Subsequent stratification of Zhejiang Province into five geographic regions (Supplementary Table 1) enabled examination of gender-specific trends from 2018 to 2024 (Supplementary Table 5). Significant declining trends were observed in male donors in eastern (Z = −3.0679, *p* = 0.002), southern (Z = −2.9715, *p* = 0.003), and western Zhejiang (Z = −1.9873, *p* = 0.047), and female donors in eastern Zhejiang (Z = −2.9285, *p* = 0.003). The overall donor population mirrored male-specific trends, showing significant reductions in eastern (Z = −3.9495, *p* < 0.001), southern (Z = −3.2526, *p* = 0.001), and western regions (Z = −2.230, *p* = 0.026; Supplementary Table 5; Figure 3A).

Age-stratified analysis of HIV positivity rate changes revealed that among both the overall donor population and male donors, only the 18-20-year-old cohort exhibited an increase in positivity rates, while all other age groups showed lower rates in 2024 compared to 2018. In contrast, female donors demonstrated universal declines across all age groups by 2024 relative to 2018 (Figure 3B).

Regional profiling of case distribution and positivity rates by gender and age identified distinct patterns. Among male donors, peak positivity occurred in the 21-25-year-old group, with the highest case burden concentrated in northern Zhejiang. Whereas among female donors, the 46–50-year-old group showed the highest positivity rate, with the largest number of cases located in eastern Zhejiang (Figure 3C). These findings underscore substantial geographic heterogeneity in HIV epidemiology across gender and age cohorts.

### Comprehensive decomposition analysis of HIV positivity trends in Zhejiang’s blood donor population

**Figure 4:**
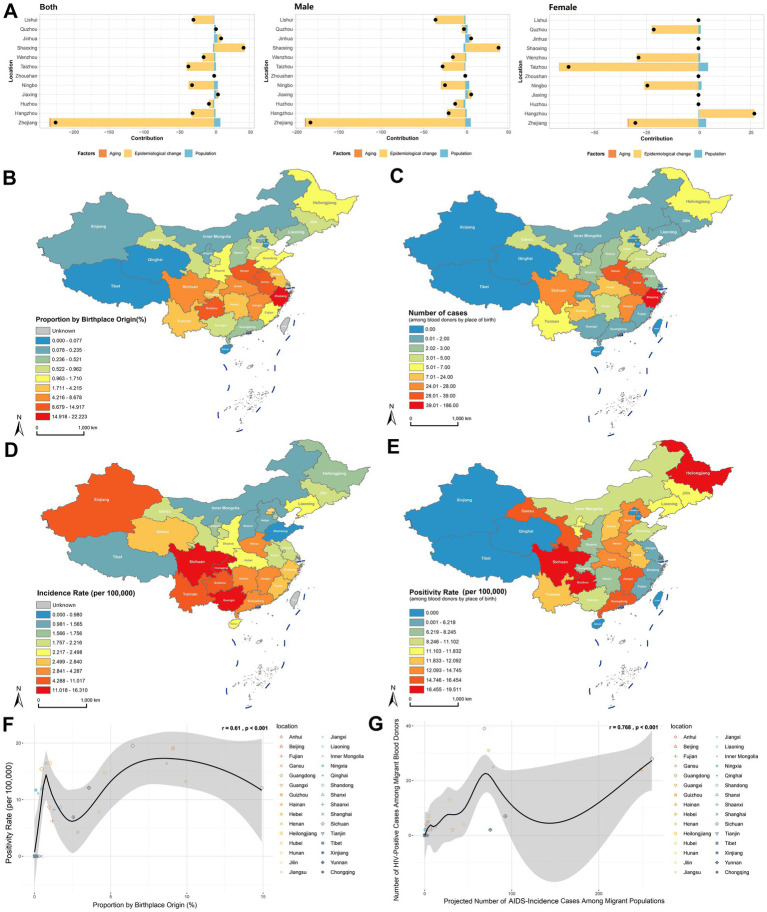
Impact of population mobility on HIV positivity rate in the blood donor population. **(A)** Decomposition analysis of HIV positivity rate for 11 prefecture-level cities and Zhejiang Province overall, including effects of population aging, population growth, and epidemiology changes adjusted for demographic characteristics. **(B)** Proportion of the population migrating into Zhejiang Province from other provinces/municipalities in China. **(C)** Number of HIV positives among blood donors by place of birth. **(D)** AIDS incidence rate by province/municipality in China, 2018–2023. **(E)** HIV positivity rate among blood donors by place of birth. **(F)** Correlation analysis between the proportion of migrants entering Zhejiang from other provinces and the HIV positivity rate among blood donors by place of birth, using Pearson correlation. **(G)** Correlation analysis between the expected number of AIDS cases based on migrants entering Zhejiang from other provinces and the number of HIV positives among blood donors by place of birth, using Pearson correlation.

Our comprehensive decomposition analysis elucidates the relative contributions of donor population aging, population growth, and demographically adjusted epidemiological changes to the evolving HIV positivity patterns across 11 prefecture-level cities and the broader Zhejiang Province. Overall, population growth contributed to the increased HIV positivity rate, while epidemiological changes played a pivotal role in driving regional disparities (Figure 4A).

As a major recipient of interprovincial migration, we further investigated how population mobility impacts HIV epidemiology among Zhejiang’s blood donors. Analysis of migration patterns using the 2020 Seventh National Population Census revealed that the intraprovincial migration rate was 22.22%, and the top three interprovincial migration sources were Anhui (14.92%), Henan (9.93%), and Guizhou (9.09%; Supplementary Table 6; Figure 4B). Native place analysis of HIV-positive donors identified that the highest case count was native Zhejiang donors (*n* = 186), whereas the top external sources were Anhui (*n* = 39), Henan (*n* = 31), and Sichuan (*n* = 28; Supplementary Table 6, Figure 4C). Cross-referencing with the China Health Statistics Yearbook (2018–2023), provinces with the highest AIDS incidence rates were Sichuan, Guangxi, Chongqing, Guizhou, Yunnan, Xinjiang, Hunan, Guangdong, Jiangxi, and Henan (Supplementary Table 6; Figure 4D). Donor native place-specific HIV positivity rates were highest among migrants from Sichuan (19.51 per 100,000), Guizhou (19.08 per 100,000), and Heilongjiang (16.48 per 100,000; Supplementary Table 6; Figure 4E).

Significant correlations were identified between migration patterns and HIV positivity in blood donors. A statistically significant association existed between the proportion of interprovincial migrants to Zhejiang (excluding native residents) and HIV positivity rate among donors by birthplace (r = 0.61, *p* < 0.001; Figure 4F). Further analysis revealed a strong correlation between projected HIV-positive cases (calculated using source province AIDS incidence rates and migration volumes) and observed HIV-positive donors by origin (r = 0.768, *p* < 0.001; Figure 4G). These findings demonstrate that population mobility significantly influences HIV positivity trends among blood donors, with migration flows directly shaping regional epidemiological profiles.

### Suppressive effect of healthcare capacity and government investment on HIV infection among blood donors, and projected HIV-positive rate trends

**Figure 5:**
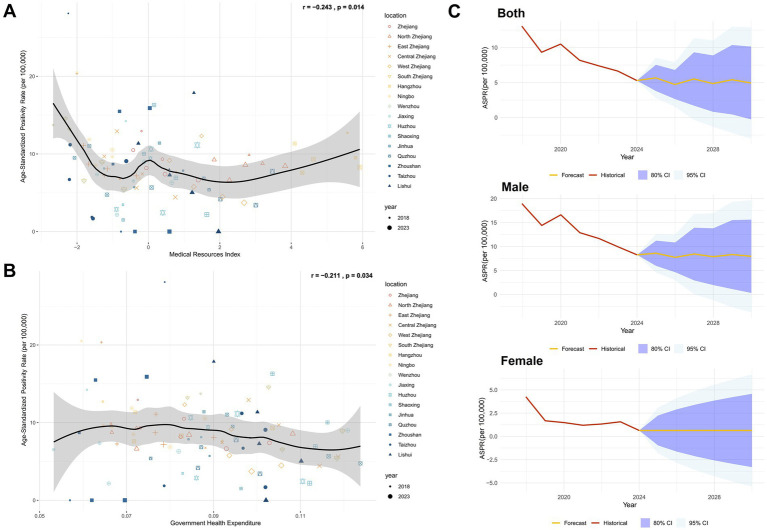
Impact of regional healthcare and government investment levels in Zhejiang on HIV positivity rate in the blood donor population and projected HIV-positive rate trends. **(A)** Correlation analysis between the regional healthcare level index and ASPR in Zhejiang Province, using Pearson correlation. **(B)** Correlation analysis between government health-related expenditure and ASPR in Zhejiang Province, using Pearson correlation. **(C)** Predicted results of the Annual Mean Incidence Rate Model (AMIRM) for HIV positivity rate in the blood donor population, 2025–2030 (See Supplementary Table 8 for detailed model fit metrics).

In addition to demographic factors, we analyzed regional healthcare capacity using metrics including hospital beds per 1,000 population, physicians per 1,000 population, nurses per 1,000 population, and healthcare technicians per 1,000 population (22). Principal Component Analysis (PCA) was applied to reduce dimensionality and generate a composite Medical Resource Index (Supplementary Table 7), and the construction and interpretation of this index, based on principal components analysis, are detailed in Supplementary Tables 7a, 7b. Correlation analysis revealed a significant inverse relationship between the Medical Resource Index and HIV positivity rate among blood donors (r = −0.243, *p* = 0.014), which indicating improved healthcare infrastructure correlates with reduced HIV prevalence (Figure 5A). Further analysis of government health expenditure ratios (Supplementary Table 7) demonstrated a significant negative correlation with donor HIV positivity rate (r = −0.211, *p* = 0.034; Figure 5B), underscoring the role of public health funding in mitigating HIV transmission risks.

Using the Autoregressive Integrated Moving Average (ARIMA) model to predict the HIV-positive rate in the blood donor population from 2025 to 2030, it was found that the HIV-positive rate in the overall and male donor populations had a fluctuating decline, the HIV-positive rate among female donors remained at current levels (Supplementary Table 8; Figure 5C).

## Discussion

This study systematically analyzed the epidemiological characteristics and influencing factors of HIV infection among blood donors in Zhejiang Province from 2017 to 2024, revealing the effectiveness and challenges of regional prevention strategies. The results demonstrated a significant overall downward trend in HIV positivity rates among blood donors in Zhejiang Province (EAPC = −12.41, *p* < 0.001), consistent with national HIV prevalence trends (21). However, substantial regional disparities were observed. While high-prevalence areas such as Taizhou and Ningbo exhibited declining rates, Shaoxing showed an upward trend, highlighting the need to address localized transmission dynamics and the adaptability of prevention measures.

Since 2016, Zhejiang Province has implemented a combined nucleic acid testing (NAT) and serological screening strategy, significantly shortening the HIV detection window period to 6–8 days (23). Data indicates that 98.44% of HIV-positive blood donors were confirmed through dual reactivity in both antigen/antibody (Ag/Ab) testing and NAT, suggesting a high probability of chronic or recent infection. Among these, 0.45% showed serological reactivity but tested negative in NAT. Notably, two Ag^+^Ab^+^NAT^−^ samples exhibited full Western blot (WB) bands, likely indicating individuals undergoing antiretroviral therapy (ART) with suppressed viral loads (24). This highlights that the widespread use of HIV treatment, which suppresses viral replication and reduces viral loads in blood, poses significant challenges for HIV detection in blood donors. It also underscores the continued importance of serological methods for HIV screening in general populations. Three Ag^−^Ab^−^NAT^+^ samples were later confirmed as seroconversion cases through follow-up, demonstrating NAT’s critical role in detecting acute-phase infections. In previous studies, the residual risk of HIV transmission through blood transfusion in Zhejiang Province was 0.266 per million donations (8), which has approached the level of developed countries. For example, the United States reported 0.5 per million donations since 2010 (25), Spain ranged from 0.52 to 2.38 cases per million donations (26), and South Korea ranged from 0.4 to 0.9 cases per million donations (27). However, vigilance remains crucial regarding potential threats to blood safety from window-period escape cases and low-level viral infections caused by antiretroviral therapy. And it is important to note that our analysis of Western Blot (WB) banding patterns has a limitation: 62.58% of confirmed positive individuals lack this data. This gap is primarily due to data-sharing barriers between the two systems. Indeed, the absence of WB data restricts in-depth investigation into the infection stage, seroconversion process, and particularly groups such as “elite controllers” or individuals with low-level viremia due to antiretroviral therapy. For example, we cannot accurately assess whether the missing data group contains a higher proportion of individuals with atypical WB bands. Therefore, it is necessary to strengthen the interoperability of information between different systems to better shield the blood supply from these low-level viral infections caused by antiretroviral therapy and thereby enhance blood safety.

The multivariate analysis revealed that male gender (OR = 7.32), non-regular blood donors (OR = 1.86), whole blood donors (OR = 3.47), and migrant populations from high-prevalence areas (OR = 1.56) were independent risk factors for HIV infection. Young males (21–25 years) exhibited the highest positivity rate, consistent with the rising infection trend among MSM (men who have sex with men) populations observed in other domestic regions (28, 29), establishing MSM transmission as a predominant pathway for HIV diffusion (30). The high prevalence among middle-aged females (46–50 years) may be associated with diversified sexual transmission routes (12). Notably, bisexual individuals within the MSM population further amplify HIV transmission risks in heterosexual contacts (11). Our multivariable analysis identified occupation as government employment as a significant protective factor (OR = 0.49). Although direct studies focusing on government employment are limited, extensive research has demonstrated that unemployment and lower socioeconomic status (SES) are key risk factors for HIV infection (31). Thus, higher SES and occupational stability among blood donors may contribute to reduced HIV risk. This protective effect is likely the result of combined behavioral factors and better healthcare access. Government employees typically receive more health education and may be more inclined to adopt safer sexual behaviors. At the same time, their stable employment environment may itself reduce exposure to high-risk situations. Moreover, in the Chinese context, government positions are generally associated with comprehensive health insurance plans, facilitating easier access to routine testing, counselling, and treatment for sexually transmitted infections, all of which are crucial components of HIV prevention.

A statistically significant association was observed between the proportion of migrants from outside Zhejiang Province (classified by birthplace) and the HIV infection rate among blood donors (r = 0.61, *p* < 0.001). Furthermore, the HIV positivity rate among migrants from other provinces (e.g., Sichuan and Guizhou) showed a strong correlation with AIDS incidence rates in their regions of origin (r = 0.768, *p* < 0.001), underscoring the impact of population mobility on regional transmission dynamics (32). However, it is important to note that these correlations are limited by potential differences in HIV testing accessibility and uptake between migrant and local populations. If migrants from high-prevalence provinces encounter barriers to testing (e.g., lack of awareness, fear of discrimination, or limited healthcare access), the true HIV prevalence among these blood donors may be underestimated in our study. This suggests that the actual strength of the association between migration and HIV risk might be stronger than what we reported. Future studies specifically designed to measure testing behaviors and true prevalence among migrant populations are needed to validate and adjust our estimates.

Seasonal scan analysis identified a higher incidence between March and July, while spatiotemporal scan analysis revealed a significant cluster of cases in March 2018 in the northeastern Zhejiang region (Hangzhou, Jiaxing, Ningbo, Taizhou, and Shaoxing). This clustering may be linked to intensified population mobility post-Spring Festival and increased high-risk behaviors during this period (16, 32). Furthermore, the monthly HIV positivity rate among blood donors in Zhejiang Province correlated significantly with the monthly AIDS incidence reported by the Chinese CDC (r = 0.445, *p* < 0.001). Decomposition analysis demonstrated that population growth contributed to increased HIV positivity rates, and regional epidemiological shifts played a pivotal role in these changes. This suggests that the overall population-level HIV prevalence is a key determinant of HIV positivity rates among blood donors. Consequently, enhanced pre-donation counseling targeting high-risk groups is warranted during the March–July high-incidence season. Further analysis revealed negative correlations between HIV positivity rates and government health expenditures (r = −0.211, *p* = 0.034) as well as the healthcare resource index (r = −0.243, *p* = 0.014). These findings highlight the importance of strengthening government-led HIV prevention and control measures. Effective strategies include public health campaigns to disseminate HIV prevention knowledge, educational initiatives for blood donors by local blood stations, and improved allocation of grassroots medical resources (e.g., hospital beds per 1,000 people, healthcare worker-to-population ratios), which may collectively reduce infection risks in blood donor populations.

The AMIRM (Analytical Model for Infectious Risk Management) prediction results indicate fluctuating HIV positivity rates among the overall population and male blood donors between 2025 and 2030, while rates among female donors are projected to remain stable. This suggests that although Zhejiang Province has achieved significant progress in HIV prevention and control, the following strategies require reinforcement:

- Blood donor management: Establish cross-regional deferral mechanisms through information systems to promptly exclude confirmed positive individuals. Strengthen donor retention efforts and encourage regular donations to increase the proportion of repeat donors, thereby enhancing blood safety (8, 9, 32).
- Targeted education: Implement interventions tailored to young males and MSM populations (33), integrating prevention campaigns in universities and communities to reduce high-risk behaviors (34).
- Resource allocation: Optimize medical investments in high-prevalence areas to improve testing capacity and follow-up coverage.

In summary, Zhejiang Province has significantly reduced HIV positivity rates among blood donors through centralized testing strategies and cross-regional deferral measures across eastern China. However, persistent challenges posed by spatiotemporal heterogeneity and population mobility demand ongoing attention. This study provides empirical evidence for optimizing regional blood safety strategies, emphasizing the integration of epidemiological surveillance, high-risk population interventions, and medical resource allocation to further reduce residual risks of HIV transmission through blood transfusion.

## Data Availability

The raw data supporting the conclusions of this article will be made available by the authors, without undue reservation.
