# Supplementary material for: Interplay of population mobility, healthcare resources, and spatiotemporal clustering: epidemiology and prevention strategies for HIV among blood donors in Zhejiang, China
Source: Front Public Health. 2025 Oct 2;13:1666694. doi: 10.3389/fpubh.2025.1666694 (PMC12529598; doi:10.3389/fpubh.2025.1666694)
Supplement: Supplementary file 1 [file Data_Sheet_1.docx]

Supplementary Material

**Supplementary Table 1. Distribution of Blood Stations and the Five Divided Regions in Zhejiang Province.**

| **Municipalities** | **Blood services** | **Regions** |
| --- | --- | --- |
| Hangzhou | Blood center of Zhejiang Province | North Zhejiang |
| Huzhou | Huzhou blood station |  |
| Jiaxing | Jiaxing blood station |  |
| Ningbo | Ningbo blood station | East Zhejiang |
| Taizhou | Taizhou blood station |  |
| Zhoushan | Zhoushan blood station |  |
| Shaoxing | Shaoxing blood station | Central Zhejiang |
| Jinhua | Jinhua blood station |  |
|  | Yiwu blood station |  |
| Wenzhou | Wenzhou blood station | South Zhejiang |
| Quzhou | Quzhou blood station | West Zhejiang |
| Lishui | Lishui blood station |  |

**Supplementary Table 2. The Standard Population in Blood Donors Used in the Study.**

| **Age group** | **Proportion** |
| --- | --- |
| 56 to 60 | 0.012589902 |
| 51 to 55 | 0.064337416 |
| 46 to 50 | 0.104387445 |
| 41 to 45 | 0.122461150 |
| 36 to 40 | 0.138555783 |
| 31 to 35 | 0.150943570 |
| 26 to 30 | 0.137750206 |
| 21 to 25 | 0.146485519 |
| 18 to 20 | 0.122489008 |

**Supplementary Table 3. The HIV burden of blood donors in Zhejiang Province in 2018 and 2024 for both sexes and all locations, with EAPC.**

| **location** | **2018** | | **2024** | | **2018-2024** | |
| --- | --- | --- | --- | --- | --- | --- |
|  | **Number of cases, No.(95%CI)** | **Age-standardised rate per100,000 population, Number of cases, No.(95%CI)** | **Number of cases, No.(95%CI)** | **Age-standardised rate per100,000 population, Number of cases, No.(95%CI)** | **Estimated annual percentage change (2018-2024), No.(95%CI)** | ***p-*value** |
| **Hangzhou** | 19 (12-30) | 12.718 (8.155-19.832) | 12 (7-21) | 8.177 (4.738-14.111) | -6.21 (-10.82 to -1.35) | 0.0552 |
| **Shaoxing** | 4 (2-10) | 8.131 (3.047-21.691) | 4 (2-10) | 11.971 (5.214-27.487) | 5.68 (-26.13 to 51.19) | 0.7747 |
| **Huzhou** | 1 (0-6) | 3.462 (0.633-18.944) | 1 (0-6) | 3.229 (0.537-19.415) | -0.14 (-24.16 to 31.48) | 0.9924 |
| **Jiaxing** | 7 (3-14) | 14.231 (6.910-29.307) | 2 (1-7) | 3.103 (0.777-12.384) | -14.67 (-31.37 to 6.09) | 0.2128 |
| **Taizhou** | 12 (7-21) | 28.105 (16.351-48.303) | 3 (1-9) | 5.523 (1.754-17.386) | -10.47 (-39.51 to 32.5) | 0.6040 |
| **Wenzhou** | 13 (8-22) | 13.720 (7.892-23.851) | 3 (1-9) | 2.791 (0.882-8.838) | -22.23 (-30.31 to -13.21) | 0.0064 |
| **Ningbo** | 17 (11-27) | 20.504 (12.779-32.898) | 3 (1-9) | 2.804 (0.880-8.934) | -21.47 (-30.78 to -10.92) | 0.0132 |
| **Quzhou** | 2 (1-7) | 7.855 (2.039-30.247) | 1 (0-6) | 4.303 (0.816-22.7) | -6.12 (-16.94 to 6.11) | 0.3586 |
| **Zhoushan** | 0 (0-1) | 0.000 (0.000-10.784) | 0 (0-1) | 0.000 (0.000-12.082) | -27.24 (-73.75 to 101.67) | 0.5677 |
| **Lishui** | 2 (1-7) | 8.030 (2.509-25.701) | 0 (0-3) | 0.000 (0.000-8.557) | -58.08 (-74.29 to -31.62) | 0.0176 |
| **Jinhua** | 4 (2-10) | 5.712 (2.125-15.35) | 3 (1-9) | 5.237 (2.007-13.663) | -8.05 (-19.6 to 5.16) | 0.2751 |
| **Zhejiang** | 81 (65-101) | 12.938 (10.407-16.085) | 32 (23-45) | 5.297 (3.803-7.377) | -12.41 (-15.24 to -9.48) | 5.00E-04 |
| **East Zhejiang** | 29 (20-42) | 20.347 (14.133-29.292) | 6 (3-13) | 3.532 (1.544-8.077) | -18.1 (-27.51 to -7.47) | 0.0238 |
| **North Zhejiang** | 27 (19-39) | 9.876 (6.796-14.35) | 15 (9-25) | 6.586 (4.033-10.756) | -4.76 (-9.19 to -0.11) | 0.1012 |
| **Central Zhejiang** | 8 (4-16) | 6.754 (3.327-13.712) | 7 (3-14) | 8.317 (4.490-15.405) | -3.35 (-15.95 to 11.13) | 0.6522 |
| **South Zhejiang** | 13 (8-22) | 13.720 (7.892-23.851) | 3 (1-9) | 2.791 (0.882-8.838) | -22.23 (-30.31 to -13.21) | 0.0064 |
| **West Zhejiang** | 4 (2-10) | 7.644 (3.032-19.267) | 1 (0-6) | 1.954 (0.369-10.357) | -22.67 (-30.67 to -13.75) | 0.0058 |

**Supplementary Table 4. Spatiotemporal analysis of HIV incidence in Zhejiang blood donors, 2018-2024.**

| **Disease Cluster Area** | **Cluster-Affected District/Country** | **Temporal Cluster（month-year）** | **RR value** | **LLR value** | ***p* value** |
| --- | --- | --- | --- | --- | --- |
| Hangzhou, | Shangcheng, Qiantang, Xihu, Binjiang, Xiaoshan, Yuhang, Linping | March 2018 | 7.86 | 19.970 | <0.001 |
| Ningbo, | Haishu, Jiangbei, Beilun, Zhenhai, Yinzhou, Xiangshan, Ninghai, Yuyao, Cixi, Fenghua |  |  |  |  |
| Jiaxing, | Nanhu, Xiuzhou, Jiashan, Haiyan, Haining, Pinghu, Tongxiang |  |  |  |  |
| Shaoxing, | Yuecheng, Keqiao, Shangyu, Xinchang, Zhuji, Shenzhou |  |  |  |  |
| Taizhou, | Jiaojiang, Huangyan, Sanmen, Tiantai, Xianju, Linhai |  |  |  |  |
| Zhoushan | Dinghai, Putuo, Daishan, Shengsi |  |  |  |  |

RR, Relative Risk; LRR, Log-likelihood Ratio.

**Supplementary Table 4a: Sensitivity Analysis of Spatiotemporal Cluster Detection using SaTScan under Different Parameter Settings**

| **Model Description** | **Temporal Cluster（month-year）** | **Locations Included** | | **Radius (km)** | **Relative Risk (RR)** | **Log-Likelihood Ratio** | ***p* value** |
| --- | --- | --- | --- | --- | --- | --- | --- |
| **Primary Model (Non-overlap, 50% threshold)** | March 2018 | **Hangzhou** | Shangcheng, **Qiantang***, Xihu, Binjiang, Xiaoshan, Yuhang, Linping | 154.88 | 7.86 | 19.970 | 0.000094 |
|  |  | **Ningbo** | **Haishu, Jiangbei,** Beilun, **Zhenhai, Yinzhou,** Xiangshan, **Ninghai,** Yuyao, Cixi, Fenghua |  |  |  |  |
|  |  | Jiaxing | Nanhu, Xiuzhou, Jiashan, Haiyan, Haining, Pinghu, Tongxiang |  |  |  |  |
|  |  | **Shaoxing** | **Yuecheng, Keqiao, Shangyu, Xinchang, Zhuji,** Shenzhou |  |  |  |  |
|  |  | **Taizhou** | Jiaojiang, Huangyan, **Sanmen, Tiantai, Xianju, Linhai** |  |  |  |  |
|  |  | Zhoushan | Dinghai, Putuo, Daishan, Shengsi |  |  |  |  |
| **Model A (Overlap, 50% threshold)** | March 2018 | **Hangzhou** | Shangcheng, **Qiantang**, Xihu, Binjiang, Xiaoshan, Yuhang, Linping | 154.88 | 7.86 | 19.970 | 0.000094 |
|  |  | **Ningbo** | **Haishu, Jiangbei,** Beilun, **Zhenhai, Yinzhou,** Xiangshan, **Ninghai,** Yuyao, Cixi, Fenghua |  |  |  |  |
|  |  | Jiaxing | Nanhu, Xiuzhou, Jiashan, Haiyan, Haining, Pinghu, Tongxiang |  |  |  |  |
|  |  | **Shaoxing** | **Yuecheng, Keqiao, Shangyu, Xinchang, Zhuji,** Shenzhou |  |  |  |  |
|  |  | **Taizhou** | Jiaojiang, Huangyan, **Sanmen, Tiantai, Xianju, Linhai** |  |  |  |  |
|  |  | Zhoushan | Dinghai, Putuo, Daishan, Shengsi |  |  |  |  |
| **Model B (Non-overlap, 40% threshold)** | March 2018 | **Hangzhou** | Shangcheng, **Qiantang**, Xihu, Binjiang, Xiaoshan, Fuyang | 100.62 | 8.33 | 18.405 | 0.00035 |
|  |  | **Ningbo** | **Haishu, Jiangbei, Zhenhai, Yinzhou, Ninghai,** Fenghua |  |  |  |  |
|  |  | **Shaoxing** | **Yuecheng, Keqiao, Shangyu, Xinchang, Zhuji** |  |  |  |  |
|  |  | **Taizhou** | **Sanmen, Tiantai, Xianju, Linhai** |  |  |  |  |
|  |  | Jinhua | Pan’an, Yiwu, Dongyang |  |  |  |  |
| **Model C (Non-overlap, 30% threshold)** | March 2018 | **Hangzhou** | **Qiantang** | 113.13 | 9.32 | 15.939 | 0.0026 |
|  |  | **Ningbo** | **Haishu, Jiangbei,** Beilun, **Zhenhai, Yinzhou,** Xiangshan, **Ninghai,** Yuyao, Cixi |  |  |  |  |
|  |  | Jiaxing | Haiyan |  |  |  |  |
|  |  | **Shaoxing** | **Yuecheng, Keqiao, Shangyu, Xinchang, Zhuji,** Shenzhou |  |  |  |  |
|  |  | **Taizhou** | Jiaojiang, **Sanmen, Tiantai, Xianju, Linhai** |  |  |  |  |
|  |  | Zhoushan | Dinghai, Putuo, Daishan |  |  |  |  |

*Bold represents regions where clustering occurs in each model.

**Supplementary Table 5. Results of the Chi-Square Trend Test on HIV Positivity Rates among Blood Donors Across Regions in Zhejiang Province, 2018-2024.**

| **Location** | **Both** | | **Male** | | **Female** | |
| --- | --- | --- | --- | --- | --- | --- |
|  | **Z score** | ***p*-value** | **Z score** | ***p*-value** | **Z score** | ***p*-value** |
| **Zhejiang** | -5.1625 | 2.437e-07 | -4.6503 | 3.314e-06 | -2.5129 | 0.01197 |
| **East Zhejiang** | -3.9495 | 7.832e-05 | -3.0679 | 0.002156 | -2.9285 | 0.003406 |
| **North Zhejiang** | -1.1622 | 0.2451 | -1.3524 | 0.1763 | 0.76001 | 0.4473 |
| **Central Zhejiang** | -0.45058 | 0.6523 | -0.77342 | 0.4393 | 0.67884 | 0.4972 |
| **South Zhejiang** | -3.2526 | 0.001144 | -2.9715 | 0.002963 | -1.0937 | 0.2741 |
| **West Zhejiang** | -2.23 | 0.02575 | -1.9873 | 0.04689 | -1.7558 | 0.07913 |

**Supplementary Table 6. Proportion of Migrated-in Population in Zhejiang, AIDS Incidence Rates, and HIV-Positive Cases/Predonation Positivity Rates among Blood Donors Born in Different Provinces.**

| **Location** | **Proportion of migrated-in population in Zhejiang(%)^@^** | **AIDS incidence rate (2018-2023) (per 100,000)^#^** | **HIV-positive blood donors by native place (2018-2024)** | |
| --- | --- | --- | --- | --- |
|  |  |  | **Number** | **Rate (per 100,000)** |
| **Anhui** | 14.92 | 1.85 | 39 | 12.09 |
| **Beijing** | 0.12 | 2.73 | 0 | 0.00 |
| **Fujian** | 1.18 | 2.64 | 2 | 6.22 |
| **Gansu** | 0.77 | 2.03 | 5 | 16.45 |
| **Guangdong** | 0.47 | 3.66 | 2 | 15.42 |
| **Guangxi** | 0.96 | 13.38 | 2 | 8.64 |
| **Guizhou** | 9.09 | 11.02 | 24 | 19.08 |
| **Hainan** | 0.07 | 2.24 | 0 | 0.00 |
| **Hebei** | 0.65 | 1.27 | 4 | 13.23 |
| **Henan** | 9.93 | 2.97 | 31 | 13.22 |
| **Heilongjiang** | 1.02 | 1.76 | 7 | 16.48 |
| **Hubei** | 4.61 | 2.49 | 13 | 14.74 |
| **Hunan** | 4.21 | 4.29 | 4 | 7.85 |
| **Jilin** | 0.53 | 2.22 | 2 | 11.81 |
| **Jiangsu** | 2.84 | 1.79 | 3 | 4.23 |
| **Jiangxi** | 8.68 | 3.66 | 25 | 16.39 |
| **Liaoning** | 0.48 | 2.42 | 2 | 11.83 |
| **Inner Mongolia** | 0.23 | 1.37 | 2 | 11.10 |
| **Ningxia** | 0.08 | 1.63 | 2 | 11.69 |
| **Qinghai** | 0.08 | 2.71 | 0 | 0.00 |
| **Shandong** | 1.71 | 0.98 | 5 | 8.66 |
| **Shanxi** | 0.52 | 1.56 | 3 | 12.08 |
| **Shaanxi** | 1.28 | 2.50 | 3 | 8.24 |
| **Shanghai** | 0.42 | 1.74 | 0 | 0.00 |
| **Sichuan** | 6.45 | 16.31 | 28 | 19.51 |
| **Tianjin** | 0.05 | 1.75 | 0 | 0.00 |
| **Tibet** | 0.01 | 1.03 | 0 | 0.00 |
| **Xinjiang** | 0.19 | 7.14 | 0 | 0.00 |
| **Yunnan** | 3.57 | 10.46 | 7 | 12.09 |
| **Zhejiang** | 22.22 | 2.84 | 186 | 6.07 |
| **Chongqing** | 2.55 | 11.91 | 2 | 6.91 |

@The proportion of the population migrating into Zhejiang Province (%) was calculated using data from the 2020 Seventh National Population Census of China, based on current residence and place of birth: (Number of people from other provinces/municipalities currently residing in Zhejiang / Total population residing in a place different from their birthplace) × 100. #The average annual AIDS incidence rate per 100,000 population for each province from 2018 to 2023 was calculated based on the China Health Statistics Yearbook (2018-2023): (Total AIDS cases in the province from 2018-2023 / Average annual resident population of the province from 2017-2022) × 100,000.

**Supplementary Table 7. Medical Resource Index and Government Health Expenditure Ratios, Zhejiang Province, from 2018 to 2023.**

| **Location** | **Year** | **Hospital beds per 1,000 population** | **Physicians per 1,000 population** | **Nurses per 1,000 population** | **Healthcare technicians per 1,000 population** | **Medical Resource Index^@^** | **Government health expenditure ratios (%)^#^** |
| --- | --- | --- | --- | --- | --- | --- | --- |
| **Hangzhou** | 2018 | 8.28 | 4.58 | 5.09 | 11.97 | 5.58 | 0.065 |
|  | 2019 | 8.27 | 4.73 | 5.31 | 12.26 | 5.80 | 0.067 |
|  | 2020 | 7.53 | 4.27 | 4.89 | 11.22 | 4.10 | 0.072 |
|  | 2021 | 7.44 | 4.51 | 5.17 | 11.66 | 4.31 | 0.072 |
|  | 2022 | 7.51 | 4.64 | 5.32 | 12.00 | 4.62 | 0.104 |
|  | 2023 | 8.00 | 5.00 | 5.78 | 12.87 | 5.92 | 0.083 |
| **Ningbo** | 2018 | 4.72 | 3.15 | 3.30 | 8.08 | -2.00 | 0.060 |
|  | 2019 | 5.08 | 3.46 | 3.68 | 8.72 | -1.02 | 0.065 |
|  | 2020 | 4.72 | 3.39 | 3.63 | 8.44 | -1.66 | 0.071 |
|  | 2021 | 4.73 | 3.59 | 3.72 | 8.71 | -1.48 | 0.072 |
|  | 2022 | 4.87 | 3.74 | 3.92 | 9.10 | -1.01 | 0.088 |
|  | 2023 | 5.19 | 3.91 | 4.14 | 9.55 | -0.26 | 0.080 |
| **Wenzhou** | 2018 | 4.59 | 3.07 | 2.96 | 7.21 | -2.67 | 0.087 |
|  | 2019 | 4.74 | 3.24 | 3.14 | 7.55 | -2.22 | 0.084 |
|  | 2020 | 4.57 | 3.28 | 3.27 | 7.73 | -2.32 | 0.103 |
|  | 2021 | 4.75 | 3.46 | 3.47 | 8.10 | -1.81 | 0.106 |
|  | 2022 | 4.91 | 3.64 | 3.67 | 8.51 | -1.30 | 0.120 |
|  | 2023 | 5.13 | 3.80 | 3.92 | 8.94 | -0.68 | 0.118 |
| **Jiaxing** | 2018 | 5.92 | 2.61 | 3.19 | 7.45 | -0.63 | 0.061 |
|  | 2019 | 5.95 | 2.80 | 3.30 | 7.69 | -0.42 | 0.053 |
|  | 2020 | 5.38 | 2.83 | 3.13 | 7.34 | -1.44 | 0.064 |
|  | 2021 | 5.42 | 3.21 | 3.51 | 8.04 | -0.88 | 0.066 |
|  | 2022 | 5.48 | 3.67 | 4.02 | 9.03 | -0.12 | 0.121 |
|  | 2023 | 5.64 | 4.00 | 4.43 | 9.85 | 0.66 | 0.082 |
| **Huzhou** | 2018 | 5.57 | 2.92 | 3.45 | 8.15 | -0.71 | 0.083 |
|  | 2019 | 5.97 | 3.08 | 3.59 | 8.44 | 0.06 | 0.089 |
|  | 2020 | 5.46 | 2.92 | 3.47 | 8.04 | -0.90 | 0.086 |
|  | 2021 | 5.89 | 3.23 | 3.74 | 8.51 | 0.07 | 0.085 |
|  | 2022 | 5.91 | 3.40 | 3.97 | 8.96 | 0.40 | 0.110 |
|  | 2023 | 6.28 | 3.70 | 4.32 | 9.56 | 1.37 | 0.095 |
| **Shaoxing** | 2018 | 5.43 | 3.11 | 3.12 | 7.61 | -1.24 | 0.087 |
|  | 2019 | 5.65 | 3.23 | 3.30 | 7.92 | -0.71 | 0.096 |
|  | 2020 | 6.05 | 3.37 | 3.25 | 7.97 | -0.13 | 0.116 |
|  | 2021 | 6.06 | 3.39 | 3.54 | 8.29 | 0.16 | 0.104 |
|  | 2022 | 6.24 | 3.63 | 3.80 | 8.82 | 0.77 | 0.113 |
|  | 2023 | 6.49 | 3.93 | 4.20 | 9.55 | 1.63 | 0.112 |
| **Jinhua** | 2018 | 5.91 | 3.08 | 3.19 | 7.98 | -0.40 | 0.089 |
|  | 2019 | 6.11 | 3.37 | 3.50 | 8.62 | 0.31 | 0.088 |
|  | 2020 | 5.04 | 2.83 | 2.96 | 7.19 | -2.07 | 0.094 |
|  | 2021 | 5.11 | 3.00 | 3.23 | 7.61 | -1.66 | 0.093 |
|  | 2022 | 5.23 | 3.19 | 3.49 | 8.07 | -1.16 | 0.124 |
|  | 2023 | 5.60 | 3.65 | 4.04 | 9.12 | 0.09 | 0.116 |
| **Quzhou** | 2018 | 6.66 | 3.30 | 3.46 | 8.34 | 0.97 | 0.084 |
|  | 2019 | 7.03 | 3.34 | 3.67 | 8.57 | 1.70 | 0.076 |
|  | 2020 | 6.96 | 3.31 | 3.62 | 8.52 | 1.55 | 0.087 |
|  | 2021 | 7.10 | 3.51 | 3.83 | 8.88 | 2.02 | 0.086 |
|  | 2022 | 7.54 | 3.67 | 4.11 | 9.44 | 3.01 | 0.101 |
|  | 2023 | 7.63 | 3.87 | 4.35 | 9.96 | 3.47 | 0.095 |
| **Zhoushan** | 2018 | 5.46 | 3.50 | 3.26 | 8.51 | -0.77 | 0.057 |
|  | 2019 | 5.34 | 3.30 | 3.30 | 8.38 | -0.99 | 0.059 |
|  | 2020 | 5.31 | 3.47 | 3.45 | 8.74 | -0.81 | 0.063 |
|  | 2021 | 5.43 | 3.62 | 3.64 | 9.14 | -0.38 | 0.062 |
|  | 2022 | 5.52 | 3.81 | 3.86 | 9.55 | 0.04 | 0.075 |
|  | 2023 | 5.70 | 3.97 | 4.09 | 9.98 | 0.59 | 0.070 |
| **Taizhou** | 2018 | 4.86 | 2.95 | 3.01 | 7.30 | -2.25 | 0.079 |
|  | 2019 | 5.07 | 3.13 | 3.32 | 7.79 | -1.58 | 0.079 |
|  | 2020 | 4.77 | 3.03 | 3.18 | 7.46 | -2.21 | 0.097 |
|  | 2021 | 4.73 | 3.08 | 3.25 | 7.56 | -2.19 | 0.096 |
|  | 2022 | 5.04 | 3.22 | 3.39 | 7.85 | -1.55 | 0.102 |
|  | 2023 | 5.40 | 3.36 | 3.70 | 8.54 | -0.62 | 0.102 |
| **Lishui** | 2018 | 6.13 | 3.65 | 3.67 | 8.95 | 0.58 | 0.098 |
|  | 2019 | 6.47 | 3.76 | 3.83 | 9.25 | 1.27 | 0.090 |
|  | 2020 | 5.70 | 3.42 | 3.54 | 8.51 | -0.28 | 0.100 |
|  | 2021 | 6.09 | 3.60 | 3.83 | 8.91 | 0.60 | 0.101 |
|  | 2022 | 6.29 | 3.78 | 4.08 | 9.45 | 1.22 | 0.110 |
|  | 2023 | 6.64 | 3.93 | 4.26 | 9.80 | 1.96 | 0.102 |
| **Zhejiang** | 2018 | 5.79 | 3.33 | 3.51 | 8.48 | -0.19 | 0.073 |
|  | 2019 | 5.99 | 3.51 | 3.76 | 8.89 | 0.39 | 0.073 |
|  | 2020 | 5.59 | 3.37 | 3.60 | 8.47 | -0.43 | 0.083 |
|  | 2021 | 5.65 | 3.56 | 3.83 | 8.85 | -0.06 | 0.082 |
|  | 2022 | 5.80 | 3.75 | 4.06 | 9.32 | 0.47 | 0.103 |
|  | 2023 | 6.13 | 4.01 | 4.41 | 10.00 | 1.40 | 0.093 |
| **North Zhejiang** | 2018 | 7.18 | 3.76 | 4.30 | 10.10 | 2.82 | 0.066 |
|  | 2019 | 7.27 | 3.94 | 4.49 | 10.41 | 3.20 | 0.067 |
|  | 2020 | 6.63 | 3.68 | 4.20 | 9.69 | 1.84 | 0.072 |
|  | 2021 | 6.66 | 3.96 | 4.51 | 10.21 | 2.27 | 0.072 |
|  | 2022 | 6.73 | 4.19 | 4.77 | 10.74 | 2.71 | 0.108 |
|  | 2023 | 7.11 | 4.53 | 5.20 | 11.56 | 3.83 | 0.084 |
| **East Zhejiang** | 2018 | 4.83 | 3.10 | 3.18 | 7.80 | -2.00 | 0.064 |
|  | 2019 | 5.10 | 3.32 | 3.51 | 8.33 | -1.23 | 0.068 |
|  | 2020 | 4.78 | 3.26 | 3.44 | 8.08 | -1.82 | 0.077 |
|  | 2021 | 4.78 | 3.40 | 3.53 | 8.30 | -1.68 | 0.077 |
|  | 2022 | 4.98 | 3.55 | 3.71 | 8.65 | -1.15 | 0.090 |
|  | 2023 | 5.30 | 3.70 | 3.97 | 9.19 | -0.34 | 0.079 |
| **Central Zhejiang** | 2018 | 5.68 | 3.09 | 3.16 | 7.80 | -0.80 | 0.088 |
|  | 2019 | 5.89 | 3.30 | 3.41 | 8.29 | -0.17 | 0.092 |
|  | 2020 | 5.47 | 3.06 | 3.08 | 7.52 | -1.24 | 0.105 |
|  | 2021 | 5.52 | 3.17 | 3.36 | 7.90 | -0.88 | 0.098 |
|  | 2022 | 5.66 | 3.38 | 3.62 | 8.39 | -0.33 | 0.119 |
|  | 2023 | 5.98 | 3.77 | 4.11 | 9.30 | 0.75 | 0.114 |
| **South Zhejiang** | 2018 | 4.59 | 3.07 | 2.96 | 7.21 | -2.67 | 0.087 |
|  | 2019 | 4.74 | 3.24 | 3.14 | 7.55 | -2.22 | 0.084 |
|  | 2020 | 4.57 | 3.28 | 3.27 | 7.73 | -2.32 | 0.103 |
|  | 2021 | 4.75 | 3.46 | 3.47 | 8.10 | -1.81 | 0.106 |
|  | 2022 | 4.91 | 3.64 | 3.67 | 8.51 | -1.30 | 0.120 |
|  | 2023 | 5.13 | 3.80 | 3.92 | 8.94 | -0.68 | 0.118 |
| **West Zhejiang** | 2018 | 6.40 | 3.47 | 3.56 | 8.64 | 0.78 | 0.092 |
|  | 2019 | 6.75 | 3.55 | 3.75 | 8.91 | 1.49 | 0.083 |
|  | 2020 | 6.30 | 3.37 | 3.58 | 8.51 | 0.59 | 0.094 |
|  | 2021 | 6.57 | 3.56 | 3.83 | 8.90 | 1.27 | 0.094 |
|  | 2022 | 6.89 | 3.73 | 4.09 | 9.45 | 2.08 | 0.106 |
|  | 2023 | 7.11 | 3.90 | 4.30 | 9.88 | 2.68 | 0.099 |

@Based on data from the Zhejiang Provincial Health Commission website (22) for 2018-2023 (including hospital beds per thousand population, doctors per thousand population, nurses per thousand population, and health technicians per thousand population), PCA was performed for dimensionality reduction to create a medical resource index. #Based on the Zhejiang Statistical Yearbook (2018-2023), we calculated the proportion of government health-related expenditure for each city, expressed as the share of health expenditure in total local fiscal expenditure (%).

**Supplementary Table 7a. Variance Explained by Principal Components**

| **Principal Component** | **Eigenvalue** | **Proportion of Variance Explained** | **Cumulative Proportion Explained** |
| --- | --- | --- | --- |
| **PC1** | 3.56 | 0.891 (89.1%) | 0.891 (89.1%) |
| **PC2** | 0.38 | 0.094 (9.4%) | 0.985 (98.5%) |
| **PC3** | 0.05 | 0.012 (1.2%) | 0.997 (99.7%) |
| **PC4** | 0.01 | 0.003 (0.3%) | 1.000 (100.0%) |

**Supplementary Table 7b. Loadings of Medical Resource Variables on Principal Components**

| **Variable** | **PC1** | **PC2** | **PC3** | **PC4** |
| --- | --- | --- | --- | --- |
| Hospital beds per 1,000 population | 0.447 | 0.871 | -0.180 | -0.095 |
| Physicians per 1,000 population | 0.504 | -0.431 | -0.712 | -0.231 |
| Nurses per 1,000 population | 0.520 | -0.189 | 0.651 | -0.520 |
| Healthcare technicians per 1,000 population | 0.525 | -0.141 | 0.192 | 0.817 |

**Supplementary Table 8. HIV Positivity Rate in Zhejiang Blood Donors from 2025 to 2030: ARIMA Model Prediction.**

| **Gender** | **Year** | **HIV Positivity Rate (per 100,000)** | **80%CI** | **95%CI** | **ARIMA Order (p,d,q)** | **AIC** | **BIC** |
| --- | --- | --- | --- | --- | --- | --- | --- |
| **Both** | 2025 | 5.666945 | 3.793423 to 7.540467 | 2.801640 to 8.532250 | (1, 1, 2) | 32.26652 | 32.05016 |
|  | 2026 | 4.726901 | 2.649055 to 6.804746 | 1.549110 to 7.904692 |  |  |  |
|  | 2027 | 5.516054 | 1.700406 to 9.331701 | -0.319476 to 11.351583 |  |  |  |
|  | 2028 | 4.853572 | 0.824780 to 8.882363 | -1.307933 to 11.015077 |  |  |  |
|  | 2029 | 5.409715 | 0.445903 to 10.373527 | -2.181780 to 13.001210 |  |  |  |
|  | 2030 | 4.942841 | -0.260044 to 10.145726 | -3.014285 to 12.899967 |  |  |  |
| **Male** | 2025 | 8.600970 | 5.966557 to 11.235380 | 4.571983 to 12.629960 | (1, 1, 2) | 36.82639 | 36.61003 |
|  | 2026 | 7.753895 | 4.655842 to 10.851950 | 3.015832 to 12.491960 |  |  |  |
|  | 2027 | 8.436736 | 2.943934 to 13.929540 | 0.036221 to 16.837250 |  |  |  |
|  | 2028 | 7.886287 | 1.965127 to 13.807450 | -1.169346 to 16.941920 |  |  |  |
|  | 2029 | 8.330013 | 1.133820 to 15.526210 | -2.675614 to 19.335640 |  |  |  |
|  | 2030 | 7.972318 | 0.327401 to 15.617240 | -3.719574 to 19.664210 |  |  |  |
| **Female** | 2025 | 0.636257 | -0.972956 to 2.245469 | -1.824822 to 3.097335 | (0, 1, 0) | 25.05257 | 24.99848 |
|  | 2026 | 0.636257 | -1.639514 to 2.912027 | -2.844234 to 4.116747 |  |  |  |
|  | 2027 | 0.636257 | -2.150982 to 3.423495 | -3.626457 to 4.898970 |  |  |  |
|  | 2028 | 0.636257 | -2.582169 to 3.854682 | -4.285901 to 5.558414 |  |  |  |
|  | 2029 | 0.636257 | -2.962053 to 4.234566 | -4.866883 to 6.139396 |  |  |  |
|  | 2030 | 0.636257 | -3.305494 to 4.578007 | -5.392130 to 6.664644 |  |  |  |

AIC, Akaike Information Criterion; BIC, Bayesian Information Criterion.

**Supplementary Figure 1. Screening Results and Western Blot Band Classification of HIV-Confirmed Positive Blood Donors.** Screening results were categorized based on the donor's antigen/antibody and nucleic acid test results. The screening-to-confirmation interval refers to the period between the blood donation date and the date of the confirmatory positive test. Western blot band reports were sourced from local CDC HIV confirmation reports. Some reports only indicated the HIV type, while 3 reports provided only HIV-1 nucleic acid quantitative results without specific band information.

**Supplementary Figure 2. Univariate and Multivariate Analysis Forest Plot of HIV-Confirmed Positive Blood Donors.**
